# Angiotensinogen and its relationship with blood pressure in young adults: the African-PREDICT study

**DOI:** 10.1038/s41371-026-01112-1

**Published:** 2026-01-27

**Authors:** Noncedo Nontando Maseko, Aletta Sophia Uys, Vuledzani Felicia Maugana, Lebo Francina Gafane-Matemane

**Affiliations:** 1 Hypertension in Africa Research Team (HART), North-West University, Potchefstroom, South Africa; 2 SAMRC Research Unit for Hypertension and Cardiovascular Disease, North-West University, Potchefstroom, South Africa

**Keywords:** Risk factors, Hypertension

## Abstract

The renin-angiotensin-aldosterone system (RAAS) plays a central role in blood pressure (BP) control through its downstream components including angiotensin II and aldosterone. However, recent data show that upstream RAAS components, such angiotensinogen, have a potential as targets for therapeutic interventions, yet remain underexplored. Our study compared BP and pulse pressure (PP) components across quartiles of circulating angiotensinogen in a healthy young population and further examined these associations after stratifying by ethnicity. The study population consisted of 1144 healthy Black and White men and women aged 20–30 years. We derived central systolic BP (SBP), central diastolic BP (DBP), central PP, and measured clinic and 24-hour (24-h) PP and BP. Angiotensinogen levels were determined in serum. Across different BP components, only nighttime PP decreased with increasing angiotensinogen quartiles (P-trend = 0.021). We found higher circulating angiotensinogen levels in White individuals as compared to their Black counterparts. After adjustments for multiple covariates in regression analyses, clinic SBP (β = 0.12, p < 0.001), clinic DBP (β = 0.10, p = 0.011), 24-h DBP (β = 0.11, p = 0.008), daytime DBP (β = 0.11, p = 0.009) and nighttime DBP (β = 0.11, p = 0.014) were positively associated with angiotensinogen only in the White group. Additional adjustment for heart rate (HR) modified these associations. In White individuals, clinic SBP (β = 0.12, p < 0.001) and clinic DBP (β = 0.08, p = 0.046) were positively associated with angiotensinogen, independent of HR, while other associations lost significance. No associations were observed in the Black group. The positive association of clinic BP with angiotensinogen in young, healthy White adults, independent of HR, suggests a probable direct role for angiotensinogen in BP regulation.

## Introduction

The renin-angiotensin-aldosterone system (RAAS) is central in blood pressure (BP) regulation [1], with several antihypertensive therapies targeting downstream RAAS to counteract the deleterious effects of angiotensin II and aldosterone [2]. However, upstream RAAS components such as angiotensinogen, a precursor of all angiotensin peptides [3], have been largely neglected in cardiovascular risk prediction, both as a substrate of RAAS activity and independent of RAAS activity. Consequently, angiotensinogen remains underresearched as a potential therapeutic target for hypertension [4].

The significance of different BP measurements in cardiovascular risk assessment is well established [5]. While clinic BP is the standard method of BP measurement and more applicable to routine care in different settings, increasing evidence suggests that central and ambulatory BP are more superior in cardiovascular risk assessment [6]. Central BP directly measures aortic pressure, providing better prediction of cardiovascular events and assessment of cardiac workload than brachial BP [7]. Ambulatory BP, which records pressure over 24 h, reveals circadian patterns such as nocturnal hypertension and non-dippers [6]. RAAS activation has been shown to elevate central, 24-hour (24-h) and clinic BP, contributing to hypertension development [8]. This further emphasises the need to include a variety of BP measurements in BP-regulation studies. Pulse pressure (PP), particularly central PP serves as a surrogate marker of arterial stiffness, providing added benefits into cardiovascular risk prediction [9]. Additionally, Fang et al. found that PP was associated with cardiovascular mortality in younger, normotensive individuals [10].

Ethnic differences in angiotensinogen levels may contribute to variations in BP regulation and hypertension prevalence across populations [11]. Exploring less studied RAAS components, like angiotensinogen in conjunction with different BP measures across different ethnic groups may enhance our understanding of hypertension risk and its underlying mechanisms. The current study aims to investigate associations of central and brachial BP and PP with angiotensinogen in apparently healthy young Black and White adults.

## Material and methods

### Study design and population

Data were collected from participants enrolled in the African Prospective study on Early Detection and Identification of Cardiovascular disease and Hypertension (African-PREDICT) study. The study included 1202 young participants at baseline, to be followed over a period of 10 years to identify novel and early markers of cardiovascular risk [12]. The inclusion criteria for this study were self-reported Black or White ethnicity, participants aged 20–30 years, and BP less than 140/90 mmHg at screening. Additionally, participants had to be human immunodeficiency virus (HIV) -uninfected, not pregnant or breastfeeding, and have no prior diagnosis or on any chronic medication including antihypertensive medication. The current sub-study used cross-sectional baseline data for 1144 participants with complete data for angiotensinogen as well as BP. All participants gave a written informed consent. The African-PREDICT study was approved by the Health Research Ethics Committee of the North-West University (NWU-00001-12-A1), complied with the Declaration of Helsinki criteria for human research and is registered on ClinicalTrials.gov (NCT03292094).

### Questionnaires

Information on age, sex, ethnicity, current use of alcohol and tobacco were obtained using the general health and demographic questionnaire. The socioeconomic status (SES) was assessed from three categories included in the questionnaire and adapted to the South African context [13]. The adapted version scored participants in three categories: skill level, education, and household income.

### Anthropometry measurements

All anthropometric measurements were performed based on the International Standards for Anthropometric Assessment [14] and included weight (SECA electronic scale, SECA, Birmingham, UK), height (SECA stadiometer, SECA, Birmingham, UK) and waist circumference (WC) (Holtain, Crymych, UK). Body mass index (BMI) was calculated using the standard weight (kg)/height(m^2^) equation.

### Blood pressure measurements

While participants were in a supine position a pulse wave analysis was performed using the SphygmoCor XCEL device (AtCor Medical Pty. Ltd., Sydney, Australia) [15]. The device estimated central SBP and PP from the brachial arterial waveform [16]. Calibration of the waveform was conducted using brachial systolic and diastolic BP. Measurements were performed in duplicate, and if two values of BP differed by more than 3 mmHg, a third measurement was performed. The two closest measurements were used to calculate an average, which was used for statistical analysis.

The Dinamap Procare BP monitor (GE Medical Systems, Milwaukee, USA) with an appropriate cuff size was used to measure clinic BP and heart rate (HR), which was taken four times, twice on each arm with the participant seated and in a relaxed state. Before the measurement of BP, the participant was requested not to have exercised, smoked, or eaten for 30 min beforehand. After a resting state of 10 min BP measurement were taken on both arms with 5 min intervals while the arm was supported at heart level. The average of the second measurement from the left and right arm was used for statistical analysis. Brachial PP was calculated as brachial systolic BP (SBP) – brachial diastolic BP (DBP).

Ambulatory BP and HR were obtained over 24-h period with the Card(X)plore devices (MediTech, Budapest, Hungary), programmed to take recordings every 30 min during the day (06:00–22:00 h) and hourly during the night (22:00–06:00 h). The device was fitted to each participant at approximately the same time each morning, using an appropriately sized cuff fitted to participant’s non-dominant arm. Percentage dipping for BP was calculated as follows: Percentage dipping = [1-(SBP_sleeping/_SBP_awake_)] *100%. 24-h PP was calculated as 24-h SBP – 24-h DBP. The percentage successful inflation rate over ambulatory period was (88.7 ± 11.16) in the total population.

### Biological sampling and biochemical analyses

Fasting blood samples were taken in the morning by a registered research nurse. Blood samples were collected and prepared according to standardised protocol and stored at −80 °C until analyses can be performed. Serum samples were analysed for creatinine, C-reactive protein (CRP), total cholesterol (TC), low-density lipoprotein cholesterol (LDL-c) and high-density lipoprotein cholesterol (HDL-c), glucose and gamma-glutamyl transferase (GGT) (Cobas Intega 400plus, Roche Basal, Switzerland). Total angiotensinogen levels were determined in serum by the Human Total Angiotensinogen Assay Kit (IBL, Japan). The assay demonstrated a sensitivity of 0.03 ng/mL and a lower limit of detection of 0.03 ng/mL. It also showed no cross-reactivity (<0.1%) with angiotensin I, II, III, IV, or other related peptides and serum proteins, ensuring high specificity for intact angiotensinogen [17]. Equilibrium angiotensin levels were quantified based on a liquid chromatography coupled to tandem mass spectrometry (LC-MS/MS) multiplex assay using 350 μL of equilibrated serum samples (Attoquant Diagnostics, Vienna, Austria). After a solid-phase-based and internal standard-controlled extraction procedure, LC-MS/MS quantification was performed using highly specific and simultaneous multiple reaction monitoring (MRM) detection of endogenous angiotensin peptides and internal standards [18]. Equilibrium angiotensin levels were used to calculate combined parameters and ratios as surrogate markers(s) for the activity of circulating RAS enzymes, including ACE-S (Ang II/Ang I) and PRA-S (Ang I + Ang II). Serum levels of cystatin-C and creatinine were used to calculate estimated glomerular filtration rate (eGFR). The eGFR calculation used the *Chronic Kidney Disease Epidemiology* (CKD-EPI) formula without the race factor [19]. Each participant collected a 24-h urine sample, starting on the day of participation. Participants were instructed to discard the first urine of the day and collect all urine passed thereafter, including the first urine of the next day. The start and finish times of urine collection were recorded. The protocol for 24-h urine collection is in accordance with the Pan American Health Organization/ World Health Organization (PAHO/WHO) [20]. Urinary sodium and potassium were measured by means of ion-selective electrode potentiometry using a Cobas Integra 400 plus (Roche, Basal, Switzerland) and used to calculate the 24-h urinary sodium/potassium (Na/K) ratio.

### Statistical analysis

Statistical analyses were performed with IBM^®^ SPSS^®^ Statistics, Version 30 (IBM Corporation, Armonk, New York). GraphPad Prism 10 was used to construct figures of comparisons between angiotensinogen as well as BP and PP components in Black and White groups. All variables were tested for normality using the Kolmogorov-Smirnov test and visual inspection of histograms and Q-Q plots prior to any further statistical analyses. Continuous data with a normal distribution was reported as the arithmetic mean ± standard deviation. Variables with a non-Gaussian distribution were logarithmically transformed and was presented as geometric mean with 5th and 95th percentile intervals. Analysis of variance (ANOVA) were performed to compare characteristics of the study population across quartiles of angiotensinogen in the total group. Comparisons were performed between the Black and White groups by using independent *t*-tests for continuous variables and the Chi-square test for categorical variables. Categorical variables (sex, ethnicity, SES class, dipping status, self-reported smoking, and alcohol use) were presented as frequencies and proportions. Analysis of covariance (ANCOVA) was performed to compare BP, PP and angiotensinogen profiles across the Black and White groups while adjusting for sex, age and SES score. Pearson and partial correlation tests were used to determine which covariates correlate with BP and angiotensinogen (Supplementary Table 2 and 3). Multiple regression analyses were performed to determine independent associations of angiotensinogen with PP and BP components in the total group as well as in the Black and White groups. All models were adjusted for the same confounding factors and covariates. Variables that were considered for inclusion are ethnicity (in the total group), age, sex, SES status score, BMI, glucose, LDL-c, CRP, self-reported smoking and alcohol intake, Na/K ratio, eGFR and HR. The HR variable included in each model matched the corresponding BP measure: clinic HR for clinic BP model, 24-h HR for 24-h BP model and central HR for central BP model. A p-value of < 0.05 was considered statistically significant.

## Results

In Table 1, we compared baseline characteristics across quartiles of angiotensinogen. A larger proportion of the highest quartile of angiotensinogen was comprised of White participants and women (all p ≤ 0.001). Nighttime PP, 24-h urinary Na/K ratio, and LDL-c decreased with increasing angiotensinogen quartiles (P-trend ≤0.021). eGFR and CRP increased with increasing angiotensinogen levels (P-trend ≤0.011).

**Table 1:** Characteristics of study population across quartiles of angiotensinogen.

Angiotensinogen range, µg/mL	Quartile 1 (n = 284) < 10.26	Quartile Q2 (n = 284) ≥ 10.26 to <15.08	Quartile 3 (n = 284) ≥ 15.08 to <23.82	Quartile 4 (n = 285) ≥ 23.82	p-value
***Demographics***					
Age, years	24.4 ± 3.09	24.6 ± 3.07	24.5 ± 3.15	24.6 ± 3.17	0.81
Sex, women/men (%)	145/139 (51.1/48.9)	126/158 (44.4/55.6)	127/157 (44.7/55.3)	192/93 (67.4/32.6)	<0.001*
Ethnicity, White/Black (%)	132/152 (46.5/53.5)	125/159 (44/56)	134/150 (47.2/52.8)	190/95 (66.7/33.3)	<0.001*
Socioeconomic status score	20.1 ± 6.14	20.8 ± 6.60	20.5 ± 6.00	21.7 ± 5.56	0.017*
Socioeconomic status					0.10
Low, n (%)	119 (41.9)	116 (41.0)	110 (38.7)	95 (33.3)	
Middle, n (%)	85 (29.9)	60 (21.2)	96 (33.8)	90 (31.6)	
High, n (%)	80 (28.2)	107 (37.8)	78 (27.5)	100 (35.1)	
***Anthropometric measurements***					
Body height, cm	168.6 ± 9.51	169.0 ± 9.82	169.1 ± 9.51	167.8 ± 9.25	0.34
Body weight, kg	70.1 ± 15.9	71.5 ± 17.5	72.8 ± 18.5	70.8 ± 17.4	0.09
Body mass index, kg/m^2^	24.6 ± 4.84	25.0 ± 5.63	25.4 ± 5.73	25.1 ± 5.83	0.46
Waist circumference, cm	78.9 ± 11.8	80.2 ± 12.6	81.5 ± 13.3	79.9 ± 12.0	0.09
***Blood pressure components***					
Clinic SBP, mmHg	116 ± 11.9	118 ± 11.2	118 ± 12.0	116 ± 11.6	**0.047**
Clinic DBP, mmHg	77 ± 7.69	79 ± 7.74	79 ± 7.66	78 ± 7.60	0.08
Clinic PP, mmHg	39 ± 8.67	39 ± 8.11	39 ± 9.03	38 ± 8.11	0.59
Heart rate, bpm	65 ± 10.6	63 ± 10.2	65 ± 10.2	67 ± 10.7	**<0.001**
Central SBP, mmHg	107 ± 9.28	109 ± 9.52	109 ± 9.46	106 ± 9.50	**<0.001**
Central DBP, mmHg	74 ± 7.60	75 ± 7.63	75 ± 7.77	73 ± 7.95	**0.007**
Central PP, mmHg	34 ± 5.75	35 ± 5.69	34 ± 5.95	33 ± 5.10	**0.001**
24-h SBP, mmHg	116 ± 9.47	117 ± 9.11	118 ± 9.74	116 ± 8.85	0.07
24-h DBP, mmHg	68 ± 5.52	69 ± 5.87	69 ± 5.90	69 ± 6.00	0.11
24-h PP, mmHg	48 ± 7.57	48 ± 7.08	49 ± 7.57	47 ± 6.88	**0.05**
Nighttime SBP, mmHg	108 ± 10.6	108 ± 10.1	109 ± 10.6	107 ± 10.1	0.22
Nighttime DBP, mmHg	59 ± 6.34	59 ± 6.87	59.9 ± 6.85	59.6 ± 6.44	0.12
Nighttime PP, mmHg	49 ± 8.28	49 ± 7.95	49 ± 8.36	47 ± 7.59	0.021*
Daytime SBP, mmHg	121 ± 9.73	122 ± 9.52	122 ± 10.1	121 ± 9.59	0.12
Daytime DBP, mmHg	73 ± 6.21	73 ± 6.36	74 ± 6.40	74 ± 6.78	0.23
Daytime PP, mmHg	48 ± 7.75	48 ± 7.40	49 ± 7.78	47 ± 6.80	0.06
Percentage dipping, %	10.6 ± 6.12	11.2 ± 5.97	11.0 ± 5.71	11.3 ± 6.19	0.46
Non-dipper, n (%)	114 (40.1)	93 (32.7)	101 (35.6)	95 (33.3)	0.66
***RAAS components***					
Angiotensinogen, µg/mL	7.25 (3.72; 10.0)	12.5 (10.4; 14.8)	18.3 (15.3; 23.0)	41.0 (24.5; 87.5)	<0.001*
PRA-S, pmol/L	86.0 (14.8; 301.7)	81.0 (12.6; 294.0)	96.9 (22.3; 335.0)	100.7 (14.1; 311.3)	**0.010**
ACE-S, pmol/L /pmol/L	2.99 (1.50; 6.18)	2.75 (1.50; 5.57)	2.80 (1.40; 5.74)	2.33 (1.00; 5.58)	**<0.001**
***Kidney function markers***					
eGFR, ml/min/1.73m^2^	119.8 ± 21.1	119.8 ± 20.5	121.4 ± 23.8	128.4 ± 25.9	<0.001*
24-h urinary Na/K ratio	3.39 (1.35; 7.26)	3.30 (1.35; 7.68)	2.95 (1.18; 6.56)	2.94 (1.20; 6.23)	0.003*
***Biochemical measurements***					
Glucose, mmol/L	5.02 (4.36; 5.75)	5.04 (4.38; 5.70)	5.04 (4.44; 5.81)	5.03 (4.38; 5.67)	0.93
C-reactive protein, mg/L	0.77 (0.08; 7.54)	0.82 (0.07; 9.27)	0.85 (0.07; 8.77)	1.12 (0.10; 11.9)	0.011*
GGT, U/L	18.4 (7.62; 52.2)	19.7 (6.96; 54.0)	20.5 (6.20; 72.8)	14.8 (5.07; 49.5)	<0.001*
Total cholesterol, mmol/L	3.81 ± 1.12	3.79 ± 1.04	3.84 ± 1.24	3.64 ± 1.38	0.20
HDL-cholesterol, mmol/L	1.21 ± 0.38	1.16 ± 0.37	1.11 ± 0.43	1.16 ± 0.49	0.08
LDL-cholesterol, mmol/L	2.4 (1.16; 4.25)	2.3 (1.19; 4.16)	2.3 (1.02; 4.24)	2.1 (0.94; 4.07)	<0.001*
***Lifestyle factors***					
Current smoker, yes n (%)	75 (26.4)	57 (20.1)	86 (30.3)	52 (18.2)	**0.002**
Current alcohol use, yes n (%)	149 (52.8)	157 (55.9)	163 (57.8)	164 (57.7)	0.60

Values are arithmetic mean ± standard deviation; geometric mean (5^th^ and 95^th^ percentile interval) for logarithmically transferred variables.

*Difference between Quartile 1 and Quartile 4. Bold text indicates p < 0.05

*n* number of participants, PRA-S angiotensin-based plasma renin activity (Ang I + Ang II), *ACE-S* angiotensin-based angiotensin-converting enzyme activity (Ang II/Ang I), *SBP* systolic blood pressure, *DBP* diastolic blood pressure, *PP* pulse pressure, *eGFR* estimated glomerular filtration rate, *HDL-* high density lipoprotein, *LDL-* low density lipoprotein, *Na/K* sodium to potassium ratio, *RAAS* renin-angiotensin-aldosterone system, *GGT* Gamma-glutamyl transferase

**Fig. 1:**
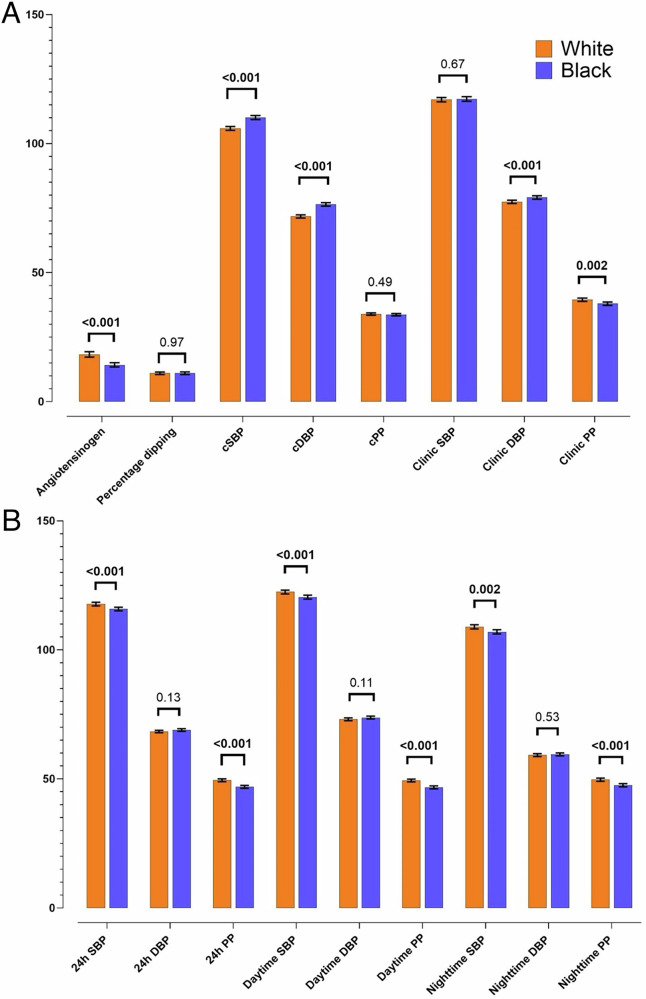
Adjusted comparisons of blood pressure, pulse pressure and angiotensinogen profiles according to ethnicity. (**A**) Angiotensinogen, percentage dipping, clinic and central blood pressure and pulse pressure and (**B**) 24 h, daytime and nighttime blood pressure and pulse pressure levels in Black and White participants. Values are mean (lower limit; upper limit); analysis of covariance used for statistical analysis. Blood pressure and pulse pressure are depicted as mmHg and angiotensinogen as µg/mL. Adjusted for sex, age and socioeconomic status score. Bold text indicates p < 0.05. *24 h* 24 h *DBP* diastolic blood pressure, *PP* pulse pressure, *SBP* systolic blood pressure.

Due to the interaction of ethnicity on association between clinic SBP and angiotensinogen (β = 0.25, p = 0.046) (Supplementary Table 1), data were stratified according to ethnicity. As presented in Table 2, there were notable differences between Black and White groups, with Black participants presenting with significantly higher central SBP, central and clinic DBP (all p < 0.001) when compared to their White counterparts. Additionally, Clinic PP (p = 0.048), 24-h SBP and PP (both p < 0.001); daytime SBP and PP (both p < 0.001); nighttime SBP and PP (p ≤ 0.018) and angiotensinogen (p < 0.001) were higher in the White group than in their Black counterparts (Table 2). These results remained robust after adjusting for sex, age and SES score (Fig. 1).

**Table 2:** The comparison of the characteristics of the study population stratified by ethnicity.

	White (n = 586)	Black (n = 558)	p-value
***Demographics***			
Age, years	24.6 ± 3.05	24.5 ± 3.18	0.48
Sex, women/men (%)	309/277 (52.7/47.3)	285/273 (51.1/48.9)	0.58
Socioeconomic status score	23.5 ± 5.35	17.9 ± 5.50	**<0.001**
Socioeconomic status			**<0.001**
Low, n (%)	117 (20.0)	325 (58.3)	
Middle, n (%)	180 (30.7)	152 (27.3)	
High, n (%)	289 (49.3)	80 (14.4)	
***Anthropometric measurements***			
Body height, cm	172.6 ± 8.82	164.4 ± 8.39	**<0.001**
Body weight, kg	76.5 ± 18.4	66.1 ± 14.5	**<0.001**
Body mass index, kg/m^2^	25.6 ± 5.40	24.6 ± 5.69	**0.002**
Waist circumference, cm	82.5 ± 13.5	77.8 ± 10.9	**<0.001**
***Blood pressure components***			
Clinic SBP, mmHg	117 ± 11.9	118 ± 11.5	0.25
Clinic DBP, mmHg	77 ± 7.39	79 ± 7.90	**<0.001**
Clinic PP, mmHg	39 ± 8.54	38 ± 8.39	**0.048**
Heart rate, bpm	67 ± 10.5	64 ± 10.3	**<0.001**
Central SBP, mmHg	106 ± 9.39	110 ± 9.11	**<0.001**
Central DBP, mmHg	72 ± 7.44	77 ± 7.31	**<0.001**
Central PP, mmHg	34 ± 5.75	34 ± 5.58	0.43
24-h SBP, mmHg	118 ± 9.60	116 ± 8.90	**<0.001**
24-h DBP, mmHg	69 ± 5.86	69 ± 5.81	0.54
24-h PP, mmHg	49 ± 7.64	47 ± 6.71	**<0.001**
Nighttime SBP, mmHg	109 ± 10.9	107 ± 9.78	**0.018**
Nighttime DBP, mmHg	59 ± 6.61	60 ± 6.66	0.19
Nighttime PP, mmHg	50 ± 8.13	48 ± 7.91	**<0.001**
Daytime SBP, mmHg	123 ± 10.1	120 ± 9.26	**<0.001**
Daytime DBP, mmHg	73 ± 6.51	73 ± 6.37	0.80
Daytime PP, mmHg	49 ± 7.82	47 ± 6.84	**<0.001**
Percentage dipping, %	11.3 ± 6.10	10.7 ± 5.88	0.09
Non-dipper, n (%)	187 (31.9)	219 (39.2)	0.052
***RAAS components***			
Angiotensinogen, µg/mL	18.3 (6.48; 71.8)	14.2 (5.57; 42.2)	**<0.001**
PRA-S, pmol/L	127.2 (39.5; 348.9)	63.8 (11.3; 274.9)	**<0.001**
ACE-S, pmol/L /pmol/L	2.69 (1.30; 5.60	2.73 (1.40; 5.90)	0.55
***Kidney function markers***			
eGFR (ml/min/1.73m^2^)	118.7 ± 25.6	126.0 ± 19.6	**<0.001**
24-h urinary Na/K ratio	2.58 (1.09; 5.33)	3.95 (1.93; 7.99)	**<0.001**
***Biochemical measurements***			
Glucose, mmol/L	5.09 (4.49; 5.77)	4.97 (4.33; 5.66)	**<0.001**
C-reactive protein, mg/L	0.79 (0.08; 8.09)	1.00 (0.09; 10.2)	**0.006**
GGT, U/L	14.9 (5.40; 47.4)	22.4 (8.48; 67.2)	**<0.001**
Total cholesterol, mmol/L	4.06 ± 1.33	3.47 ± 0.97	**<0.001**
HDL-cholesterol, mmol/L	1.17 ± 0.46	1.14 ± 0.38	0.24
LDL-cholesterol, mmol/L	2.46 (1.02; 4.43)	2.06 (0.99; 3.70)	**<0.001**
***Lifestyle factors***			
Current smoker, yes n (%)	86 (30.3)	52 (18.2)	0.21
Current alcohol use, yes n (%)	163 (57.8)	164 (57.7)	0.91

Values are arithmetic mean ± standard deviation; geometric mean (5^th^ and 95^th^ percentile interval) for logarithmically transferred variables. Bold text indicates p < 0.05.

*n* number of participants, PRA-S angiotensin-based plasma renin activity (Ang I + Ang II), *ACE-S* angiotensin-based angiotensin-converting enzyme activity (Ang II/Ang I), *SBP* systolic blood pressure, *DBP* diastolic blood pressure, *PP* pulse pressure, *eGFR* estimated glomerular filtration rate, *HDL-* high density lipoprotein, *LDL-* low density lipoprotein, *Na/K* sodium to potassium ratio, *RAAS* renin-angiotensin-aldosterone system, *GGT* Gamma-glutamyl transferase.

In the total group (Supplementary Table 4), clinic SBP, 24-h DBP, daytime DBP and nighttime DBP (all p ≤ 0.046) associated positively with angiotensinogen. However, these results were in fact driven by the White ethnicity as evident after group stratification. Clinic DBP (β = 0.10, p = 0.024), 24-h DBP (β = 0.11, p = 0.007), daytime DBP (β = 0.10, p = 0.015) and nighttime DBP (β = 0.09, p = 0.027) associated positively with angiotensinogen (Table 3). Additionally, clinic SBP associated positively with angiotensinogen (β = 0.12, p < 0.001) in the White group only.

**Table 3:** Independent associations of blood pressure and pulse pressure components with angiotensinogen across ethnicity stratification.

	White participants (n = 586)		Black participants (n = 558)	
	Angiotensinogen, µg/mL			
	Adj. R^2^	$\beta$ (95% Cl)	Adj. R^2^	$\beta$ (95% Cl)
Central SBP, mmHg	0.33	0.03 (−0.04; 0.10)	0,12	0.02 (−0.09; 0.11)
Central DBP, mmHg	0.12	0.06 (−0.03; 0.14)	0.00	0.01 (−0.10; 0.11)
Central PP, mmHg	0.27	−0.02 (−0.10; 0.05)	0.18	0.03 (−0.07; 0.13)
Clinic SBP, mmHg	**0.42**	**0.12 (0.05; 0.19)**‡	0.21	0.01 (−0.08; 0.11)
Clinic DBP, mmHg	**0.16**	0.09 (0.01; 0.17)*	0.08	−0.01 (−0.12; 0.10)
24-h SBP, mmHg	0.52	0.05 (−0.01; 0.11)	0.32	0.01 (−0.08; 0.10)
24-h DBP, mmHg	**0.19**	**0.11 (0.03; 0.19)**†	0.10	−0.00 (−0.11; 0.10)
Daytime SBP, mmHg	0.50	0.06 (−0.01; 0.12)	0.30	0.02 (−0.06; 0.11)
Daytime DBP, mmHg	**0.17**	0.10 (0.02; 0.18)*	0.09	−0.01 (−0.12; 0.09)
Nighttime SBP, mmHg	0.38	0.03 (−0.04; 0.10)	0.24	0.00 (−0.09; 0.09)
Nighttime DBP, mmHg	**0.12**	0.09 (0.01; 0.18)*	0.06	0.03 (−0.08; 0.14)

Multiple regression analyses models included age, sex, socioeconomic status score, body mass index, glucose, low density lipoprotein-cholesterol, C-reactive protein, self-reported smoking and alcohol intake, 24-h sodium/potassium ratio and estimated glomerular filtration rate, with each BP component as a dependent variable in each model. Bold indicates p < 0.05.

*DBP* diastolic blood pressure, *PP* pulse pressure, *SBP* systolic blood pressure.

‡ p < 0.001.

† p < 0.01; *p < 0.05

### Sensitivity analyses

To determine the possible effect of HR on the association between circulating angiotensinogen and BP in Black and White participants, we additionally adjusted for HR. As a result, previously observed associations between angiotensinogen and 24-h, daytime and nighttime DBP in the White group became non-significant (p ≥ 0.93) except for the association with clinic SBP (Adj. R^2^ = 0.42, β = 0.12, p < 0.001) and clinic DBP (Adj. R^2^ = 0.19, β = 0.08. p = 0.046), which remained significant in the White group only (Supplementary Table 6, Supplementary Table 7).

## Discussion

We investigated the associations of central and peripheral BP and PP with angiotensinogen in young, apparently healthy adults. In the overall group, angiotensinogen positively associated with clinic BP, 24-h, daytime and nighttime DBP. After stratification by ethnicity, these associations were observed in the White group only. Interestingly, HR modified the associations of angiotensinogen with 24-h, daytime and nighttime DBP in the White group. Clinic BP remained significantly associated with angiotensinogen, independent of HR.

Ethnicity has long been recognised as an important factor influencing RAAS function, hypertension development and response to antihypertensive medication [21]. Unlike renin, the rate limiting factor of RAAS, which is known to be lower in Black populations [22], contradictory findings have been reported regarding angiotensinogen levels among different ethnic groups. In a recent multi-ethnic study of 5786 participants aged 45 to 84 years, angiotensinogen levels were elevated in the White population compared to the Black population [11]. This is consistent with the current study where we observed elevated levels of angiotensinogen in White participants compared to their Black counterparts. However, a study by Bloem et al. [23] found that Black adolescents, with an average age of 14.8 years, had elevated serum levels of angiotensinogen compared to their White counterparts. It was speculated that this may be attributed to the higher BMI in their Black participants compared to their White counterparts. In the current study, measures of adiposity including BMI and WC were increased in the White group compared to their Black counterparts. It is known that adipose tissue increases the production of circulating angiotensinogen levels [24]. Therefore, the high circulating angiotensinogen levels in the White participants may be attributed to in part, adipose tissue.

In the current study, we found significant associations between angiotensinogen and both clinic SBP and DBP. Importantly, these associations were independent of HR. These findings suggest that RAAS activation and its impact on clinic BP are maintained in part by angiotensinogen, which may to an extent be independent of the autonomic nervous system activity, which largely regulates HR [25]. To the best of our knowledge, this is the first study to observe an association between circulating angiotensinogen and clinic BP, independent of HR in young adults. While previous studies have observed associations between angiotensinogen and clinic BP, these associations were seen in individuals of African ancestry [26], amongst an older cohort [27] and/or when HR was not accounted for as a confounder.

In a sub-study of the African-PREDICT, Botha et al. [28] observed positive correlations between clinic BP and measures of subclinical organ damage. When considered with the findings of the current study, clinic BP may reflect not only hemodynamic pressure but also underlying vascular factors that may influence cardiovascular health in young adults.

The autonomic nervous system includes two anatomical divisions [25]. The sympathetic nervous system (SNS) which increases HR, and the parasympathetic system that suppresses HR [25]. The RAAS is known to impact cardiovascular homeostasis through direct actions on the peripheral blood vessels and through modulation of the autonomic nervous system [29]. In our initial analysis, we observed a positive association between angiotensinogen with clinic and ambulatory DBP in the overall study population and among White participants. However, upon adjustment for HR, the observed associations between angiotensinogen and ambulatory DBP were no longer statistically significant. These results might be explained by the complementary actions of the RAAS and the SNS on SBP and DBP [30]. The main effector peptide of RAAS, angiotensin II acts through the angiotensin type 1 receptor (AT1R) to increase sympathetic flow and BP [29]. The observed results may suggest that the effects of angiotensinogen on ambulatory BP, particularly DBP may be through RAAS and SNS interplay.

Of interest was the lack of associations between all BP and PP components (central, clinic and 24-h) and circulating angiotensinogen among the Black participants. In line with our findings, a Jamaican study involving normotensive individuals (average age 44 years) and hypertensive individuals (average age 54 years) found no association between angiotensinogen levels and both clinic SBP and DBP [31]. The absence of an association between all BP and PP components and angiotensinogen in Black individuals of the present study therefore aligns with existing evidence that upstream RAAS components have limited influence on BP regulation in individuals of African ancestry [22]. Individuals of African ancestry commonly exhibit low plasma renin activity and are more likely to experience salt-sensitive, volume-dependent hypertension [8, 22]. As a result, upstream RAAS components such as renin and angiotensinogen may be suppressed and play a limited, if any pathophysiological role in hypertension, which could possibly explain the lack of BP- angiotensinogen associations in the Black population of the current study. Furthermore, antihypertensive medication targeting the RAAS especially angiotensinogen which are currently of main interest [3], might be less effective in populations of the African ancestry.

We expected positive associations between angiotensinogen and central BP and all PP components (central, brachial, and 24-h), however no associations were found. Central BP and PP are closely associated with arterial stiffness, wave reflection and the load experienced by the heart, kidneys and large arteries [32]. The absence of an association in this cohort may be due to the compliant nature of the large arteries of young, healthy individuals [33, 34].

Our study should be interpreted within the context of its limitations and strengths. This is a cross-sectional study therefore causality should not be inferred. The study population was recruited from a specific region in South Africa and is not representative of all Black and White populations. The results remained robust after multiple adjustments; however, we cannot rule out residual confounding factors. The current literature supports the association between angiotensinogen gene variants and BP outcomes, while evidence regarding circulating angiotensinogen and its relationship with BP remains sparse. As a result, we were unable to make extensive comparisons with previous studies, which may restrict the contextualisation of our findings within the broader literature. Central BP was estimated using the SphygmoCor XCEL device. However, recent validation work by Schultz et al. reported that this device does not meet the Artery Society accuracy criteria when compared with invasive measurements [35]. This limitation in the method may have influenced our results, specifically the absence of any association between central BP and angiotensinogen. The strengths of the study are that it included a relatively large sample size with young, healthy Black and White adults with detailed phenotypes, including angiotensinogen and BP profiles. To the best of our knowledge this is the first study to investigate the relationship between angiotensinogen and multiple components of BP and PP in a young healthy population.

In conclusion, we found higher circulating angiotensinogen in White individuals as compared to the Black participants. In this young population, HR modified the associations between angiotensinogen and 24-h BP, while clinic BP associated with angiotensinogen independent of HR. This highlights the potential direct and indirect role of angiotensinogen in BP regulation.

## Summary

### What is known about the topic

- The role of RAAS effects on BP is well-known, primarily through the effects of angiotensin II.
- The association between genetic variants of angiotensinogen and BP mostly in older, unhealthy populations have been reported.

### What this study adds

- This is the first study to report the relationship of circulating angiotensinogen and different BP components and; -
- observe increased levels of angiotensinogen in young healthy White participants.
- A comprehensive understanding of upstream RAAS activity involvement in pressure regulation by including all BP profiles, independent of HR.

## Supplementary information

Supplementary tables
