# Supplementary material for: Angiotensinogen and its relationship with blood pressure in young adults: the African-PREDICT study
Source: J Hum Hypertens. 2026 Jan 27;40(3):209–16. doi: 10.1038/s41371-026-01112-1 (PMC13002465; doi:10.1038/s41371-026-01112-1)
Supplement: Supplementary file 1 — Supplementary tables [file 41371_2026_1112_MOESM1_ESM.docx]

**Supplementary tables**

**Supplementary Table 1: Interaction terms of ethnicity on the associations between blood pressure and pulse pressure components and angiotensinogen**

|  | **Total group**  **(n=1144)** | |
| --- | --- | --- |
|  | **Angiotensinogen*ethnicity** | |
|  | Adj. R^2^ | β (95% Cl) |
| Central SBP, mmHg | 0.27 | 0.11 (-0.15; 0.37) |
| Central DBP, mmHg | 0.15 | 0.17 (-0.12; 0.45) |
| Central PP, mmHg | 0.23 | -0.09 (-0.36; 0.18) |
| Clinic SBP, mmHg | **0.33** | **0.25 (0.00; 0.50)*** |
| Clinic DBP, mmHg | 0.13 | 0.27 (-0.02; 0.55) |
| Clinic PP, mmHg | 0.28 | 0.08 (-0.18; 0.34) |
| 24-h SBP, mmHg | 0.44 | 0.09 (-0.14; 0.32) |
| 24-h DBP, mmHg | 0.15 | 0.26 (-0.02; 0.54) |
| 24-h PP, mmHg | 0.41 | -0.07 (-0.31; 0.16) |
| Daytime SBP, mmHg | 0.42 | 0.08 (-0.15; 0.32) |
| Daytime DBP, mmHg | 0.13 | 0.27 (-0.02; 0.55) |
| Daytime PP, mmHg | 0.38 | -0.12 (-0.36; 0.12) |
| Nighttime SBP, mmHg | 0.33 | 0.07 (-0.18; 0.32) |
| Nighttime DBP, mmHg | 0.09 | 0.11 (-0.18; 0.40) |
| Nighttime PP, mmHg | 0.33 | 0.01 (-0.24; 0.26) |
| Percentage dipping, mmHg | 0.02 | -0.04 (-0.34; 0.26) |

**Supplementary Table 2: Pearson correlations of blood pressure and pulse pressure components with angiotensinogen across ethnicity stratification**

|  | **Angiotensinogen, µg/mL** | | | |
| --- | --- | --- | --- | --- |
|  | **White participants**  **(n=586)** | | **Black participants**  **(n=558)** | |
|  | *r* | *p* | *r* | *p* |
| Clinic SBP, mmHg | 0.01 | 0.91 | -0.01 | 0.85 |
| Clinic DBP, mmHg | 0.05 | 0.27 | 0.01 | 0.79 |
| Clinic PP, mmHg | -0.03 | 0.42 | -0.01 | 0.75 |
| Central SBP, mmHg | -0.05 | 0.28 | 0.01 | 0.89 |
| Central DBP, mmHg | 0.05 | 0.27 | 0.01 | 0.91 |
| Central PP, mmHg | **-0.14** | **<0.001** | 0.01 | 0.79 |
| 24-h SBP, mmHg | -0.05 | 0.21 | -0.01 | 0.85 |
| 24-h DBP, mmHg | 0.08 | 0.05 | 0.02 | 0.63 |
| 24-h PP, mmHg | **-0.12** | **0.005** | -0.03 | 0.49 |
| Daytime SBP, mmHg | -0.04 | 0.32 | 0.00 | 0.92 |
| Daytime DBP, mmHg | 0.08 | 0.07 | 0.01 | 0.88 |
| Daytime PP, mmHg | **-0.12** | **0.006** | -0.00 | 0.96 |
| Night-time SBP, mmHg | -0.05 | 0.19 | -0.02 | 0.59 |
| Night-time DBP, mmHg | 0.06 | 0.16 | 0.05 | 0.25 |
| Night-time PP, mmHg | **-0.12** | **0.004** | -0.07 | 0.10 |
| Percentage dipping, % | 0.04 | 0.40 | 0.05 | 0.25 |

|  | **Angiotensinogen, µg/mL** | | | |
| --- | --- | --- | --- | --- |
|  | **White participants**  **(n=586)** | | **Black participants**  **(n=558)** | |
|  | *r* | *p* | *r* | *p* |
| Clinic SBP, mmHg | **0.16** | **<0.001** | 0.01 | 0.81 |
| Clinic DBP, mmHg | **0.12** | **0.004** | -0.02 | 0.70 |
| Clinic PP, mmHg | **0.09** | **0.031** | 0.04 | 0.37 |
| Central SBP, mmHg | 0.03 | 0.47 | -0.01 | 0.91 |
| Central DBP, mmHg | 0.08 | 0.07 | -0.00 | 0.93 |
| Central PP, mmHg | -0.07 | 0.11 | 0.01 | 0.81 |
| 24-h SBP, mmHg | **0.09** | **0.040** | 0.01 | 0.80 |
| 24-h DBP, mmHg | **0.14** | **<0.001** | -0.01 | 0.84 |
| 24-h PP, mmHg | -0.02 | 0.66 | 0.02 | 0.61 |
| Daytime SBP, mmHg | **0.10** | **0.030** | 0.03 | 0.53 |
| Daytime DBP, mmHg | **0.13** | **0.002** | -0.02 | 0.72 |
| Daytime PP, mmHg | -0.02 | 0.64 | 0.05 | 0.28 |
| Night-time SBP, mmHg | 0.05 | 0.21 | -0.01 | 0.88 |
| Night-time DBP, mmHg | **0.10** | **0.012** | 0.02 | 0.64 |
| Night-time PP, mmHg | -0.03 | 0.47 | -0.03 | 0.48 |
| Percentage dipping, % | 0.02 | 0.56 | 0.05 | 0.23 |

**Supplementary Table 3: Partial correlations of blood pressure and pulse pressure components with angiotensinogen across ethnicity stratification**

**Supplementary Table 4: Independent associations of blood pressure and pulse pressure components with angiotensinogen in the total group**

|  | **Total group**  **(n=1144)** | |
| --- | --- | --- |
|  | **Angiotensinogen, µg/mL** | |
|  | Adj. R^2^ | β (95% Cl) |
| Central SBP, mmHg | 0.27 | 0.04 (-0.02; 0.09) |
| Central DBP, mmHg | 0.15 | 0.05 (-0.02; 0.11) |
| Central PP, mmHg | 0.23 | -0.00 (-0.06; 0.06) |
| Clinic SBP, mmHg | **0.32** | **0.07 (0.01; 0.12)*** |
| Clinic DBP, mmHg | 0.12 | 0.60 (-0.00; 0.12) |
| Clinic PP, mmHg | 0.28 | 0.40 (-0.02; 0.10) |
| 24-h SBP, mmHg | 0.44 | 0.04 (-0.02; 0.09) |
| 24-h DBP, mmHg | **0.14** | **0.08 (0.011; 0.14)*** |
| 24-h PP, mmHg | 0.41 | -0.01 (-0.06; 0.04) |
| Daytime SBP, mmHg | 0.42 | 0.04 (-0.01; 0.04) |
| Daytime DBP, mmHg | **0.13** | **0.06 (0.00; 0.13)*** |
| Daytime PP, mmHg | 0.38 | 0.00 (-0.05; 0.05) |
| Nighttime SBP, mmHg | 0.33 | 0.02 (-0.30; 0.77) |
| Nighttime DBP, mmHg | **0.09** | **0.08 (0.01; 014)*** |
| Nighttime PP, mmHg | 0.33 | -0.04 (-0.09; 0.02) |
| Percentage dipping, mmHg | 0.02 | 0.03 (-0.03; 0.10) |

**Supplementary Table 5: Heart rate-independent associations of blood pressure and pulse pressure components with angiotensinogen in the total group**

|  | **Total group**  **(n=1144)** | |
| --- | --- | --- |
|  | **Angiotensinogen, µg/mL** | |
|  | Adj. R^2^ | β (95% Cl) |
| Central SBP, mmHg | 0.23 | -0.01 (-0.06; 0.05) |
| Central DBP, mmHg | 0.14 | -0.00 (-0.07; 0.06) |
| Central PP, mmHg | 0.28 | -0.01 (-0.06; 0.05) |
| Clinic SBP, mmHg | **0.32** | **0.06 (0.01; 0.12)*** |
| Clinic DBP, mmHg | 0.13 | 0.03 (-0.03; 0.09) |
| Clinic PP, mmHg | 0.29 | 0.06 (0.00; 0.11) |
| 24-h SBP, mmHg | 0.44 | 0.04 (-0.01; 0.09) |
| 24-h DBP, mmHg | 0.24 | 0.06 (-0.00; 0.11) |
| 24-h PP, mmHg | 0.42 | 0.01 (-0.04; 0.06) |
| Daytime SBP, mmHg | 0.43 | 0.05 (-0.00; 0.10) |
| Daytime DBP, mmHg | 0.24 | 0.04 (-0.01; 0.10) |
| Daytime PP, mmHg | 0.40 | 0.02 (-0.03; 0.07) |
| Nighttime SBP, mmHg | 0.34 | 0.03 (-0.03; 0.08) |
| Nighttime DBP, mmHg | **0.16** | **0.07 (0.01; 0.13)*** |
| Nighttime PP, mmHg | 0.33 | -0.02 (-0.08; 0.03) |
| Percentage dipping, mmHg | 0.04 | 0.03 (-0.03; 0.10) |

**Supplementary Table 6: Heart rate-independent associations of blood pressure and pulse pressure components with angiotensinogen across ethnicities**

|  | **White participants**  **(n=586)** | | **Black participants**  **(n=558)** | |
| --- | --- | --- | --- | --- |
|  | **Angiotensinogen, µg/mL** | | | |
|  | Adj. R^2^ | β (95% Cl) | Adj. R^2^ | β (95% Cl) |
| Central SBP, mmHg | 0.34 | 0.02 (-0.05; 0.10) | 0,12 | 0.01 (-0.09; 0.11) |
| Central DBP, mmHg | 0.21 | 0.03 (-0.05; 0.10) | 0.02 | 0.01 (-0.09; 0.12) |
| Central PP, mmHg | 0.34 | 0.00 (-0.07; 0.08) | 0.22 | 0.02 (-0.07; 0.12) |
| Clinic SBP, mmHg | **0.42** | **0.12 (0.05; 0.19)**‡ | 0.22 | 0.01 (-0.07; 0.12) |
| Clinic DBP, mmHg | **0.19** | **0.08 (0.00; 0.15)*** | 0.09 | -0.01 (-0.11; 0.10) |
| 24-h SBP, mmHg | 0.53 | 0.04 (-0.02; 0.10) | 0.32 | 0.02 (-0.07; 0.10) |
| 24-h DBP, mmHg | 0.35 | 0.07 (0.00; 0.14) | 0.15 | 0.01 (-0.09; 0.11) |
| Daytime SBP, mmHg | 0.52 | 0.05 (-0.02; 0.11) | 0.31 | 0.03 (-0.06; 0.11) |
| Daytime DBP, mmHg | 0.34 | 0.06 (-0.01; 0.14) | 0.14 | -0.00 (-0.10; 0.10) |
| Nighttime SBP, mmHg | 0.39 | 0.03 (-0.05; 0.10) | 0.24 | 0.00 (-0.09; 0.09) |
| Nighttime DBP, mmHg | 0.19 | 0.07 (-0.01; 0.15) | 0.08 | 0.04 (-0.07; 0.14) |

**Supplementary Table 7: Full models of associations between clinic blood pressure and angiotensinogen across ethnicities**

|  | **White participants**  **(n=586)** | **Black participants**  **(n=558)** | **White participants**  **(n=586)** | **Black participants**  **(n=558)** |
| --- | --- | --- | --- | --- |
|  | **Clinic SBP, mmHg** | | **Clinic DBP, mmHg** | |
|  | β (95% Cl) | β (95% Cl) | β (95% Cl) | β (95% Cl) |
| Age, years | 0.01 (-0.06; 0.09) | -0.01 (-0.09; 0.08) | 0.05 (-0.04; 0.14) | **0.22 (0.12; 0.32)** ‡ |
| Sex, men and women | **1.12 (0.96; 1.28)**‡ | **0.98 (-0.76; 1.21)**‡ | **0.64 (0.47; 0.82)** ‡ | **0.53 (0.28; 0.78)** ‡ |
| Socioeconomic status score | **0.01 (-0.01; 0.02)** | **-0.01 (-0.03; 0.01)** | 0.02 (0.00; 0.03) | 0.00 (-0.02; 0.02) |
| Body mass index, kg/m^2^ | **0.30 (0.22; 0.39)**‡ | **0.23 (0.12; 0.34)**‡ | **0.20 ( 0.11; 0.29)** ‡ | 0.10 (-0.02; 0.23) |
| Angiotensinogen, µg/mL | **0.12 (0.05; 0.19)**‡ | 0.01 (-0.08; 0.10) | **0.08 (0.00; 0.15)*** | -0.01 (-0.12; 0.10) |
| eGFR, ml/min/1.73m^2^ | -0.01 (-0.09; 0.07) | -0.07 (-0.18; 0.05) | -0.08 (-0.17; 0.01) | -0.11 (-0.24; 0.02) |
| 24-h urinary Na/K ratio | 0.06 (-0.01; 0.13) | -0.00 (-0.10; 0.09) | -0.02 (-0.09; 0.06) | 0.70 (-0.04; 0.18) |
| Glucose, mmol/L | 0.03 (-0.05; 0.11) | 0.08 (-0.01; 0.16) | 0.06 (-0.02; 0.15) | 0.05 (-0.04; 0.15) |
| C-reactive protein, mg/L | -0.06 (-0.14; 0.03) | -0.02 (-0.13; 0.08) | -0.02 (-0.12; 0.07) | -0.06 (-0.17; 0.06) |
| LDL- cholesterol, mmol/L | -0.08 (-0.17; 0.01) | -0.04 (-0.13; 0.06) | -0.12 (-0.22; -0.03) | -0.02 (-0.12; 0.09) |
| Heart rate, bpm | 0.04 (-0.03; 0.11) | -0.02 (-0.12; 0.08) | **0.23 (0.15; 0.32)** ‡ | 0.14 (0.02; 0.25) |
| Alcohol, yes and no | 0.00 (-0.13; 0.14) | 0.09 (-0.08; 0.26) | 0.06 (-0.09; 0.21) | 0.07 (-0.13; 0.26) |
| Smoking, yes and no | -0.05 (-0.22; 0.11) | -0.13 (-0.35; 0.08) | -0.03 (-0.21; 0.16) | 0.02 (-0.22; 0.26) |

**Supplementary Table legends**

**Supplementary Table 1: Interaction terms of ethnicity on the associations between blood pressure and pulse pressure components and angiotensinogen.** Multiple regression analyses models included age, ethnicity, sex, socioeconomic status score, body mass index, angiotensinogen, glucose, low density lipoprotein-cholesterol, C-reactive protein, self-reported smoking and alcohol intake, 24-h sodium/potassium ratio and estimated glomerular filtration rate. Angiotensinogen depicted as µg/mL and ethnicity is Black and White. Bold indicates p<0.05. *24-h* 24-hour *DBP* diastolic blood pressure, *PP* pulse pressure, *SBP* systolic blood pressure.

**Supplementary Table 2: Pearson correlations of blood pressure and pulse pressure components with angiotensinogen across ethnicity stratification.** *24-h* 24-hour *DBP* diastolic blood pressure, *PP* pulse pressure, *SBP* systolic blood pressure, *r* correlation coefficient, *p* significance value. Bold text indicates *p*<0.05

**Supplementary Table 3: Partial correlations of blood pressure and pulse pressure components with angiotensinogen across ethnicity stratification.** Adjusted for age, sex, body mass index. *24-h* 24-hour *DBP* diastolic blood pressure, *PP* pulse pressure, *SBP* systolic blood pressure, *r* correlation coefficient, *p* significance value. Bold text indicates p<0.05.

**Supplementary Table 4: Independent associations of blood pressure and pulse pressure components with angiotensinogen in the total group.** Linear multiple regression analyses models included age, sex, ethnicity, socioeconomic status score, body mass index, glucose, low density lipoprotein-cholesterol, C-reactive protein, self-reported smoking and alcohol intake, 24h sodium/potassium ratio, and estimated glomerular filtration rate. Bold indicates p<0.05. ‡ p <0.001; † p<0.01; *p <0.05. *24-h* 24-hour *DBP* diastolic blood pressure, *PP* pulse pressure, *SBP* systolic blood pressure

**Supplementary Table 5: Heart rate-independent associations of blood pressure and pulse pressure components with angiotensinogen in the total group.** Linear multiple regression analyses models included age, sex, ethnicity, socioeconomic status score, body mass index, glucose, low density lipoprotein-cholesterol, c-reactive protein, self-reported smoking and alcohol intake, 24h sodium/potassium ratio, heart rate and estimated glomerular filtration rate. Bold indicates p<0.05. ‡ p <0.001; † p<0.01; *p <0.05. *24-h* 24-hour *DBP* diastolic blood pressure, *PP* pulse pressure, *SBP* systolic blood pressure

**Supplementary Table 6: Heart rate-independent associations of blood pressure and pulse pressure components with angiotensinogen across ethnicities.** Multiple regression analyses models included age, sex, socioeconomic status score, body mass index, glucose, low density lipoprotein-cholesterol, c-reactive protein, self-reported smoking and alcohol intake, 24-h sodium/potassium ratio, heart rate and estimated glomerular filtration rate. Bold indicates p<0.05. ‡ p <0.001; † p<0.01; *p <0.05. *24-h* 24-hour *DBP* diastolic blood pressure, *PP* pulse pressure, *SBP* systolic blood pressure

**Supplementary Table 7: Full models of associations between clinic blood pressure and angiotensinogen across ethnicities.** Linear multivariable regression analyses models included age, sex, socioeconomic status score, body mass index, glucose, low density lipoprotein-cholesterol, c-reactive protein, self-reported smoking and alcohol intake, 24-h sodium/potassium ratio, heart rate and estimated glomerular filtration rate. Both clinic SBP and DBP are dependant variables. Bold indicates p<0.05. ‡ p <0.001; *p <0.05. *24-h* 24-hour *DBP* diastolic blood pressure, *PP* pulse pressure, *SBP* systolic blood pressure *Na/K* sodium: potassium ratio *LDL* low density lipoprotein
